# Avaliação do Seguimento de 1 Ano dos Pacientes Incluídos no Registro da Prática Clínica em Pacientes de Alto Risco Cardiovascular (REACT)

**DOI:** 10.36660/abc.20190885

**Published:** 2020-12-07

**Authors:** Pedro Gabriel Melo de Barros e Silva, Otavio Berwanger, Dalton Bertolim Precoma, Margaret Assad Cavalcante, José Fernando Vilela-Martin, Estêvão Lanna Figueiredo, Renato Delascio Lopes, Luiz Carlos Bodanese, Jorge Ilha Guimarães, Jadelson Pinheiro de Andrade, Angelo Amato Vincenzo de Paola, Marcus Vinicius Bolivar Malachias, Luiz Alberto Piva e Mattos, Fernando Bacal, Oscar Pereira Dutra

**Affiliations:** 1 Instituto de Pesquisa HCor Sao PauloSP Brasil Instituto de Pesquisa HCor, Sao Paulo, SP - Brasil; 2 Hospital Samaritano Paulista Sao PauloSP Brasil Hospital Samaritano Paulista, Sao Paulo, SP - Brasil; 3 Hospital Israelita Albert Einstein Sao PauloSP Brasil Hospital Israelita Albert Einstein, Sao Paulo, SP - Brasil; 4 Pontificia Universidade Católica do Paraná Escola de Medicina CuritibaPR Brasil Pontificia Universidade Católica do Paraná - Escola de Medicina, Curitiba, PR - Brasil; 5 Sociedade Hospitalar Angelina Caron Campina Grande do SulPR Brasil Sociedade Hospitalar Angelina Caron – Cardiologia, Campina Grande do Sul, PR - Brasil; 6 Universidade do Oeste Paulista Presidente PrudenteSP Brasil Universidade do Oeste Paulista (Unoeste), Presidente Prudente, SP – Brasil; 7 Hospital Regional de Presidente Prudente Presidente PrudenteSP Brasil Hospital Regional de Presidente Prudente, Presidente Prudente, SP - Brasil; 8 Faculdade de Medicina de São José do Rio Preto São José do Rio PretoSP Brasil Faculdade de Medicina de São José do Rio Preto (FAMERP), São José do Rio Preto, SP - Brasil; 9 Departamento de Hipertensão Arterial Sociedade Brasileira de Cardiologia Rio de JaneiroRJ Brasil Departamento de Hipertensão Arterial da Sociedade Brasileira de Cardiologia, Rio de Janeiro, RJ - Brasil; 10 Hospital Lifecenter Belo HorizonteMG Brasil Hospital Lifecenter, Belo Horizonte, MG - Brasil; 11 Duke University Hospital DurhamNorth Carolina EUA Duke University Hospital, Durham, North Carolina - EUA; 12 Hospital São Lucas Porto AlegreRS Brasil Hospital São Lucas, Porto Alegre, RS - Brasil; 13 Sociedade Brasileira de Cardiologia Rio de JaneiroRJ Brasil Sociedade Brasileira de Cardiologia, Rio de Janeiro, RJ - Brasil; 14 Hospital da Bahia SalvadorBA Brasil Hospital da Bahia, Salvador, BA - Brasil; 15 Universidade Federal de São Paulo Escola Paulista de Medicina São PauloSP Brasil Universidade Federal de São Paulo Escola Paulista de Medicina, São Paulo, SP - Brasil; 16 Faculdade de Ciências Médicas de Minas Gerais Belo HorizonteMG Brasil Faculdade de Ciências Médicas de Minas Gerais, Belo Horizonte, MG - Brasil; 17 Instituto de Hipertensão Arterial Belo HorizonteMG Brasil Instituto de Hipertensão Arterial - Diretoria Clínica, Belo Horizonte, MG - Brasil; 18 Rede D’or de Hospitais São PauloSP Brasil Rede D’or de Hospitais, São Paulo, SP - Brasil; 19 Universidade de São Paulo Faculdade de Medicina Hospital das Clínicas São PauloSP Brasil Universidade de São Paulo Faculdade de Medicina Hospital das Clínicas Instituto do Coração, São Paulo, SP - Brasil; 20 Instituto de Cardiologia Fundação Universitária de Cardiologia do Rio Grande do Sul Porto AlegreRS Brasil Instituto de Cardiologia – Fundação Universitária de Cardiologia do Rio Grande do Sul, Porto Alegre, RS – Brasil

**Keywords:** Doenças Cardiovasculares, Fatores de Risco, Medicamentos Sob Prescrição, Estudos Multicêntricos como Assunto, Registro Médico Coordenado

## Abstract

**Fundamento:**

Na prática clínica, há evidências de falhas na prescrição de terapias baseadas em evidências para pacientes de alto risco cardiovascular. Entretanto, no Brasil, ainda são insuficientes os dados sobre a evolução ao longo de 1 ano desses pacientes.

**Objetivos:**

Descrição no acompanhamento de 12 meses da utilização de terapias baseadas em evidência e da ocorrência de desfechos cardiovasculares maiores e seus principais preditores em um registro brasileiro multicêntrico de pacientes de alto risco cardiovascular.

**Métodos:**

Estudo observacional prospectivo que documentou a prática clínica ambulatorial de indivíduos acima de 45 anos e de alto risco cardiovascular tanto em prevenção primária como secundária. Os pacientes foram seguidos por 1 ano e avaliou-se a prescrição de terapias baseadas em evidência e a ocorrência de eventos cardiovasculares maiores (infarto agudo do miocárdio [IAM], acidente vascular cerebral [AVC], parada cardíaca e mortalidade por causa cardiovascular). Valores de p < 0,05 foram considerados estatisticamente significantes.

**Resultados:**

De julho de 2010 até agosto de 2014, 5.076 indivíduos foram incluídos em 48 centros, sendo 91% dos 4.975 pacientes elegíveis acompanhados em centros de cardiologia e 68,6% em prevenção secundária. Em 1 ano, o uso concomitante de antiplaquetários, estatinas e inibidores da enzima conversora de angiotensina (IECA) reduziu de 28,3% para 24,2% (valor de p < 0,001). A taxa de eventos cardiovasculares maiores foi de 5,46%, e os preditores identificados foram: idade, pacientes em prevenção secundária e nefropatia diabética.

**Conclusões:**

Neste grande registro nacional de pacientes de alto risco cardiovascular, foram identificados preditores de risco semelhantes aos registros internacionais, porém a adesão da prescrição médica a terapias baseadas em evidência esteve abaixo dos dados da literatura internacional e apresentou piora significativa em 1 ano. (Arq Bras Cardiol. 2020; [online].ahead print, PP.0-0)

## Introdução

As doenças cardiovasculares habitualmente são manifestações decorrentes de um substrato aterosclerótico arterial.^1 - 4^ Em conjunto, acometem mais de 4% da população mundial e suas complicações agudas, conhecidas como eventos cardiovasculares, constituem a principal causa de morte e incapacidade globalmente em ambos os sexos.^2 - 4^ No Brasil, assim como em outros países em desenvolvimento, a frequência dessas doenças permanece aumentando ao longo dos anos, o que reforça a necessidade de melhor compreensão da evolução desses pacientes na prática clínica dessas regiões.^2 - 7^

Apesar da elevada morbimortalidade, diversas estratégias para reduzir o risco de complicações nesses pacientes têm sido desenvolvidas.^8 - 12^ Dentre essas opções, os pacientes de alto risco cardiovascular podem se beneficiar de terapias antitrombóticas (antiplaquetários), estatinas e IECA ou bloqueadores dos receptores de angiotensina (BRA).^8 - 12^ Contudo, a aplicação dessas terapias na prática clínica tem se mostrado insuficiente, especialmente em países em desenvolvimento.^13 - 15^ No Brasil, publicação dos dados parciais do registro da prática clínica em pacientes com alto risco cardiovascular (REACT) mostrou que o uso combinado de antiplaquetários, estatinas e IECA foi identificado em apenas 34% dessa população.^15^ Apesar da relevância desses dados, há limitações nessa análise, pois tais informações sobre a adesão da prescrição médica de terapias baseadas em evidência foram coletadas de forma transversal, e as mudanças no seguimento prospectivo não foram reportadas até o momento. Ademais, há ainda necessidade de idenificar, em uma população brasileira de indivíduos de alto risco cardiovascular, qual a real taxa de eventos esperada e quais os preditores associados a tais eventos.

O objetivo do presente estudo é avaliar ao longo de 12 meses, em pacientes de alto risco cardiovascular atendidos em centros brasileiros, a proporção de pacientes que recebem de forma continuada intervenções com benefício comprovado e os fatores associados à evolução clínica tardia, particularmente sobre a taxa de ocorrência de eventos cardiovasculares maiores durante o seguimento.

## Métodos

O registro REACT representa um projeto de documentação do atendimento real do paciente de alto risco cardiovascular em centros de todas as regiões brasileiras, incluindo hospitais públicos e privados, bem como unidades básicas de saúde.

### Delineamento e Execução do Estudo

O Registro REACT é um projeto da Sociedade Brasileira de Cardiologia (SBC), cuja operação foi realizada pelo Instituto de Pesquisa do Hospital do Coração (IP-HCor) e cujos métodos foram previamente publicados.^15 , 16^ De maneira resumida, trata-se de pesquisa obervacional, prospectiva, voluntária e multicêntrica, cuja inclusão de pacientes ocorreu de julho de 2010 até agosto de 2014 em 48 unidades de saúde que incluíram hospitais públicos e hospitais privados, bem como Unidades Básicas de Saúde (UBS). Houve abrangência dos centros nas cinco regiões brasileiras com a seguinte distribuição: Sudeste (45,8%), Norte (6,3%), Nordeste (14,6%), Sul (29,2%) e Centro-Oeste (4,2%). A seleção de centros foi realizada por convites abertos a centros interessados via SBC e pelo próprio centro coordenador (IP-HCor). O estudo foi iniciado após aprovação do Comitê de Ética em Pesquisa e os dados foram coletados após consentimento individual dos pacientes. As informações da análise transversal visando documentar a prática clínica do manejo de pacientes de alto risco cardiovascular em território nacional já foram previamente publicadas.^15^ Adicionalmente, foi realizado seguimento longitudinal desses pacientes em 6 e 12 meses com os seguintes objetivos: aferir a adesão da prescrição médica às recomendações de terapias baseadas em evidências, avaliar a ocorrência de eventos cardiovasculares maiores e identificar seus respectivos preditores.

### Participantes do Estudo

De forma resumida, os participantes do estudo deveriam apresentar idade superior a 45 anos e pelo menos um dos seguintes fatores:^15 , 16^ 1) qualquer evidência clínica de doença arterial (coronariana, cerebrovascular ou arterial periférica); 2) diabetes melito (DM); 3) três fatores de risco cardiovascular, excetuando-se DM: hipertensão arterial sistêmica (HAS), tabagismo, dislipidemia, idade superior a 70 anos, nefropatia diabética, história familiar de doença arterial coronariana (DAC), doença carotídea assintomática (subclínica). O primeiro grupo que já tinha doença arterial conhecida foi considerado como pacientes já em uma fase de prevenção secundária, independentemente da presença dos demais critérios de inclusão. Em relação aos demais participantes, eles foram considerados como prevenção primária com DM (segundo critério de inclusão) ou sem DM (aqueles incluídos apenas pelo terceiro critério de inclusão). Como se trata de estudo de prática clínica com critérios pragmáticos, os critérios de exclusão foram: recusa em fornecer Termo de Consentimento Livre e Esclarecido (TCLE), condição psiquiátrica ou neurocognitiva que impeça a obtenção de dados clínicos fidedignos (definida pelo julgamento dos investigadores); e expectativa de vida menor que 6 meses.

### Procedimentos do Estudo e Variáveis Analisadas15,16

A coleta de dados ocorreu na internação com dados basais (visita índice) e também em duas visitas de seguimento clínico aos 6 e 12 meses para aferição da adesão da prescrição médicas às recomendações de terapias baseadas em evidências e para avaliar a ocorrência de eventos cardiovasculares maiores. Essas visitas de seguimento poderiam ocorrer de forma presencial na rotina assistencial ou por telefone. Tendo a característica de um estudo pragmático, a identificação de comorbidade dos pacientes (p. ex., HAS, dislipidemia) poderiam ser realizadas da seguinte forma: relato pelo paciente, uso de medicamento (anti-hipertensivo, hipolipemiante) ou avaliação do investigador (neste último, os centros foram orientados a seguirem as recomendações de critérios diagnósticos adotadas pelas diretrizes vigentes da SBC). Foram coletadas informações sobre a prescrição de medicamentos para avaliar a adesão da prescrição médica às recomendações baseadas em evidência. O esquema terapêutico baseado em evidência que foi considerado no registro REACT estava de acordo com diretrizes vigentes.^8 - 12^ Não foram coletadas informações sobre o uso efetivo do medicamento pelos pacientes.

### Desfechos do Estudo

Conforme descrito na publicação de métodos do estudo,^16^ o desfecho primário foi relacionado com a prescrição de intervenções com benefício comprovado (como, por exemplo, ácido acetilsalicílico, estatinas, IECA) e o impacto na evolução clínica tardia. Os desfechos clínicos avaliados na evolução clínica tardia incluíram: IAM, AVC, parada cardíaca e mortalidade total e por causa cardiovascular.^15 , 16^ Esses desfechos foram reportados pelo investigador, sem utilização de um comitê independente para adjudicação de eventos.

### Análise Estatística

Avaliação de normalidade de distribuição de variáveis contínuas foi realizada por meio de histogramas. Variáveis contínuas de distribuição normal foram descritas como média ± desvio padrão. Variáveis categóricas foram descritas por frequências absolutas e relativas, as proporções foram comparadas pelo teste de Qui-quadrado ou o Teste (exato) de Fisher-Freeman-Halton. A identificação de preditores independentes de eventos combinados (óbito, IAM, parada cardiorrespiratória ou AVC) foi realizada por modelos de risco proporcionais de Cox, tendo em vista que foram coletadas informações sobre as datas dos eventos. Essa análise de preditores foi realizada inicialmente de forma univariada, avaliando os seguintes fatores: idade, sexo, histórico de DAC, IAM prévio, histórico de AVC/AIT, história de DAP, DM, HAS, nefropatia diabética, tabagismo, doença carotídea assintomática e uso combinado de antiplaquetário, estatina e IECA no *baseline* ; as variáveis com valor de p <0,15 foram incluídas na análise multivariada. Valores de p apresentados são do tipo bilateral, e p < 0,05 é considerado estatisticamente significante nas análises finais. As suposições de proporcionalidade de cada variável preditora e global foram verificadas por meio dos resíduos de Schoenfeld padronizados.^17^ Modelos de Equações de Estimação Generalizada (EEG) foram utilizados para avaliar a terapia medicamentosa ao longo do tempo. Todas as análises foram realizadas com auxílio do programa R versão 3.6.1.

## Resultados

Entre julho de 2010 até agosto de 2014, 5.076 pacientes foram recrutados nesse registro nacional. Contudo, excluindo-se pacientes sem informação de elegibilidade e sem informações de *baseline* , restaram 4.975 pacientes para análise sendo 91% deles acompanhados em centros de cardiologia ( Tabela 1 ). Em um total de 407 pacientes (8,2%), não foi possível obter informação alguma de 12 meses (perda de seguimento).

**Tabela 1:** – Caracteristicas basais

Características basais	Total (n = 4.975)
Idade; média ± DP	65,4 ± 10 (n = 4.975)
Sexo (Masculino)	2.614/4.975 (52,5%)
Etnia	
Branca	3.422/4.975 (68,8%)
Negra	571/4.975 (11,5%)
Asiática	76/4.975 (1,5%)
Parda	900/4.975 (18,1%)
Indígena	6/4.975 (0,1%)
Tipo de Centro	
Cardiologia	4.505/4.950 (91%)
Neurologia	7/4.950 (0,1%)
Cirurgia vascular	3/4.950 (0,1%)
Endocrinologia	114/4.950 (2,3%)
Medicina interna	99/4.950 (2%)
Atenção primária	222/4.950 (4,5%)
Prevenção	
Primária	428/4.975 (8,6%)
Primária DM	1.135/4.975 (22,8%)
Secundária	3.412/4.975 (68,6%)
IMC; média ± DP	28,5 ± 5,2 (n = 4.959)
IMC ≥ 25	3.660/4.959 (73,8%)
DAC	2.867/4.975 (57,6%)
Infarto agudo do miocárdio prévio	1.510/4.975 (30,4%)
AVC	710/4.975 (14,3%)
DAP	799/4.975 (16,1%)
DM	2.814/4.975 (56,6%)
Múltiplos fatores de risco (ao menos três fatores)	3.057/4.975 (61,4%)
Hipertensão	4.451/4.975 (89,5%)
Dislipidemia	3.638/4.975 (73,1%)
Nefropatia diabética	406/4.975 (8,2%)
Idade superior a 70 anos	1.700/4.975 (34,2%)
Tabagismo atual	515/4.975 (10,4%)
Histórico familiar de DAC	2.478/4.975 (49,8%)
Doença carotídea assintomática	605/4.975 (12,2%)
Pressão arterial	
Sistólica	132,3 ± 19,7 (n = 4.975)
Diastólica	79,5 ± 11,4 (n = 4.975)
Exames laboratoriais	
Colesterol total (mg/dL)	178 ± 58,5 (n = 3.041)
LDL (mg/dL)	99,6 ± 39 (n = 2.834)
HDL (mg/dL)	45,4 ± 14,4 (n = 2.996)
Triglicérides (mg/dL)	159,8 ± 116,3 (n = 3.049)
Glicemia (mg/dL)	126,7 ± 55,2 (n = 3.327)
Hemoglobina glicosilada (%)	7,2 ± 2,1 (n = 1.953)
Creatinina (mg/dL)	1,1 ± 0,8 (n = 3.305)

*DP: desvio padrão; DM: Diabetes melito; IMC: índice de massa corporal; DAC: doença arterial coronariana; AVC: acidente vascular cerebral; DAP: doença periférica; HDL: lipoproteína de alta densidade; LDL: lipoproteína de baixa densidade.*

### Características Basais

O perfil clínico dos pacientes evidenciou que a idade média foi de 65,4 (± 10), 52,5% do sexo masculino e 68,6% eram pacientes em prevenção secundária ( Tabela 1 ). Dentre os diagnósticos de doença cardiovascular estabelecida, doença coronária foi a mais comum e esteve presente em quase 60% da amostra ( Tabela 1 ).

## Adesão da Prescrição Médica a Terapias Baseadas em Evidência

**Figura 1:**
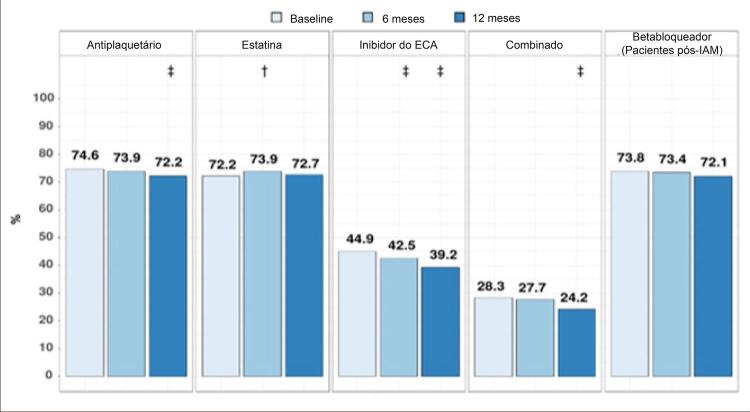
– Prescrição de terapias para prevenção cardiovascular de acordo com o tempo de seguimento. Para a comparação da continuidade da prescrição do medicamento nos seguimentos com o baseline, foi ajustado um modelo de equações de estimação generalizada (EEG) para dados binários para considerar a dependência entre as observações. ‡ Valor de p < 0,001; comparação entre o seguimento e o baseline. † Valor de p < 0,01; comparação entre o seguimento e o baseline. * Valor de p < 0,05; comparação entre o seguimento e o baseline.

Dentre os pacientes incluídos, 74,6% utilizavam antiplaquetários; 72,2%, estatinas; e 42,5%, IECA ( Tabela 2 ). O percentual variou conforme o critério de inclusão, sendo maior no grupo de prevenção secundária em que o uso de antiplaquetário e o uso de estatina ficou próximo de 80% ( Tabela 2 ). Dentre os pacientes com história de IAM, 73,8% recebiam betabloqueadores na avaliação basal. No seguimento, o uso concomitante de antiplaquetários, estatinas e IECA reduziu de 28,3% para 24,2% (valor de P < 0,001), sendo a redução mais evidente nos usários de IECA ( Figura 1 ).

**Tabela 2:** –Uso de terapias para prevenção cardiovascular e controle dos fatores de risco de acordo com características da população

	Primária (n = 428)	Primária DM (n = 1.135)	Secundária (n = 1.733)	Secundária e DM (n = 1.679)	Total (n = 4.975)	p
**Medicação (Baseline)**						
Antiplaquetário	225/428 (52,6%)	731/1.135 (64,4%)	1403/1.733 (81%)	1.354/1.679 (80,6%)	3.713/4.975 (74,6%)	<0,001
Estatina	276/428 (64,5%)	720/1.135 (63,4%)	1347/1.733 (77,7%)	1.249/1.679 (74,4%)	3.592/4.975 (72,2%)	<0,001
IECA	171/428 (40%)	519/1.135 (45,7%)	758/1.733 (43,7%)	787/1.679 (46,9%)	2.235/4.975 (44,9%)	0,043
Combinação	64/428 (15%)	263/1.135 (23,2%)	527/1.733 (30,4%)	554/1.679 (33%)	1.408/4.975 (28,3%)	<0,001
Betabloqueador (Paciente com IAM)			607/816 (74,4%)	507/694 (73,1%)	1.114/1.510 (73,8%)	-
Diurético tiazídico (Pacientes com hipertensão)	174/387 (45%)	555/1038 (53.5%)	496/1.481 (33,5%)	642/1.545 (41,6%)	1.867/4.451 (41,9%)	<0,001
**Controle dos fatores de risco (baseline)**						
Hemoglobina glicada						
< 7%	146/150 (97,3%)	321/655 (49%)	361/408 (88,5%)	292/740 (39,5%)	1.120/1.953 (57,3%)	<0,001
7% a 8%	1/150 (0,7%)	144/655 (22%)	22/408 (5,4%)	150/740 (20,3%)	317/1.953 (16,2%)	
≥ 8%	3/150 (2%)	190/655 (29%)	25/408 (6,1%)	298/740 (40,3%)	516/1.953 (26,4%)	
Glicemia						
< 100 mg/dL	185/284 (65,1%)	137/838 (16,3%)	664/1.074 (61,8%)	236/1.131 (20,9%)	1.222/3.327 (36,7%)	<0,001
100 a 125 mg/dL	90/284 (31,7%)	268/838 (32%)	342/1.074 (31,8%)	310/1.131 (27,4%)	1.010/3.327 (30,4%)	
≥ 126 mg/dL	9/284 (3,2%)	433/838 (51,7%)	68/1.074 (6,3%)	585/1.131 (51,7%)	1.095/3.327 (32,9%)	
Pressão						
< 130/80 mmHg	274/428 (64%)	582/1.135 (51,3%)	1.066/1.733 (61,5%)	904/1.679 (53,8%)	2.826/4.975 (56,8%)	<0,001
130/80 a 139/89 mmHg	97/428 (22,7%)	322/1.135 (28,4%)	432/1.733 (24,9%)	466/1.679 (27,8%)	1.317/4.975 (26,5%)	
≥ 140/90 mmHg	57/428 (13,3%)	231/1.135 (20,4%)	235/1.733 (13,6%)	309/1.679 (18,4%)	832/4.975 (16,7%)	
LDL						
< 50 mg/dL	1/269 (0,4%)	40/712 (5,6%)	53/939 (5,6%)	93/914 (10,2%)	187/2.834 (6,6%)	<0,001
50 a 69 mg/dL	25/269 (9,3%)	97/712 (13,6%)	145/939 (15,4%)	173/914 (18,9%)	440/2.834 (15,5%)	
≥ 70 mg/dL	243/269 (90,3%)	575/712 (80,8%)	741/939 (78,9%)	648/914 (70,9%)	2.207/2.834 (77,9%)	

*Valor de p (Teste Qui-Quadrado) < 0,05 indica que a terapia para prevenção/fator de risco é dependente da característica da população. DM: Diabetes melito; IAM: infarto agudo do miocárdio; IECA: inibidor da enzima conversora de angiotensina; LDL: lipoproteína de baixa densidade.*

## Controle de Fatores de Risco

No geral, 16,7% dos pacientes apresentaram pressão arterial ≥ 140×90 mmHg. Na avaliação laboratorial basal, identificou-se que o controle da hemoglobina glicada < 7% em 47,5% dos diabéticos, sendo mais frequente o controle nos pacientes em prevenção primária. O nível de LDL-colesterol esteve acima de 70 mg/dL em 76,6% dos pacientes, e mais de 90% dos pacientes em prevenção secundária apresentaram LDL-colesterol > 50 mg/dL. Dentre os pacientes sem diagnóstico prévio de HAS e/ou DM ,17,9% (94/524) apresentaram níveis de pressão arterial ≥ 140×90, 3,6% (77/2.161) apresentaram glicemia de jejum ≥ 126 e 4,1% (88/2.161) hemoglobina (Hb) glicada ≥ 6,5%. De forma combinada, dentre os pacientes sem diagnóstico prévio de HAS ou DM, 10,3% (247/2.392) apresentaram níveis patológicos de pressão arterial e/ou glicemia.

As orientações para medidas não farmacológicas foram reportadas em cerca de 80% das vistas, sendo semelhante na prevenção primária e secundária para cessação de tabagismo, mas maior na prevenção primária para atividade física e dieta cardioprotetora.

## Desfechos Clínicos

Em 12 meses, a mortalidade global (cardiovascular ou não) foi de 4,92%, sendo maior na região Nordeste (9,33%; IC 95% 6,1%-12,6%) seguida pelas regiões Centro-Oeste (8,6%; IC 95% 3,0%-14,1%), Sul (4,9%; IC 95% 3,7%-6,1%) e Sudeste (4,3%; IC 95% 3,5%-5,1%). A análise da região Norte foi prejudicada pela baixa inclusão (99 pacientes), com 30% de perda de seguimento, tendo sido reportado apenas um óbito (1,5%; IC 95% 0,0%-4,3%).

**Figura 2:**
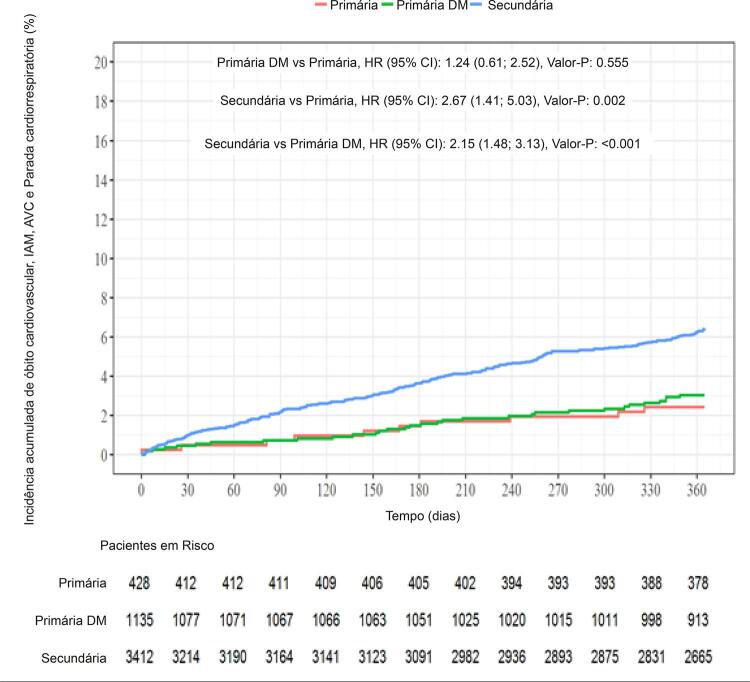
– Taxa de eventos em 1 ano de acordo com critério de inclusão. IAM: infarto agudo do miocárdio; AVC: acidente vascular cerebral; DM: diabetes melito.

A taxa de eventos cardiovasculares maiores na população total foi de 5,46 por 100 pacientes-ano no grupo de prevenção secundária ( Figura 2 ) e os preditores identificados para eventos cardiovasculares foram idade, prevenção secundária e nefropatia diabética ( Tabela 3 ).

**Tabela 3:** – Fatores preditores de risco cardiovascular. Análises univariada e multivariada

Variáveis	Análise univariada		Análise multivariada	

	HR [95% IC]	Valor de p	HR [95% IC]	Valor de p
Idade (incremento de 1 ano)	1,036 [1,025; 1,047]	<0,001	1,035 [1,024; 1,046]	<0,001
Sexo (masculino)	1,123 [0,900; 1,401]	0,303	-	-
História de DAC (sim)	1,686 [1,329; 2,139]	<0,001	1,324 [0,989; 1,772]	0,059
IAM prévio (sim)	1,672 [1,338; 2,090]	<0,001	1,515 [1,155; 1,988]	0,003
História de AVC/AIT (sim)	1,738 [1,335; 2,263]	<0,001	1,481 [1,132; 1,938]	0,004
História de DAP (sim)	1,951 [1,520; 2,503]	<0,001	1,651 [1,271; 2,143]	<0,001
DM (sim)	1,191 [0,951; 1,492]	0,127	1,227 [0,967; 1,557]	0,093
Hipertensão (sim)	0,829 [0,593; 1,159]	0,272	-	-
Nefropatia diabética (sim)	1,826 [1,324; 2,518]	<0,001	1,438 [1,021; 2,025]	0,037
Fumante (sim)	0,950 [0,656; 1,376]	0,785	-	-
Doença carotídea assintomática (sim)	1,008 [0,724; 1,404]	0,963	-	-
Medicamento combinado (sim)*	1,083 [0,852; 1,377]	0,513	-	-

*Medicamento combinado: uso combinado de antiplaquetário, estatina e IECA no baseline. DAC: doença arterial coronariana; IAM: infarto agudo do miocárdio; AVC: acidente vascular cerebral; DAP: doença periférica; AIT: ataque isquêmico transitório; DM: Diabetes melito.*

## Discussão

O registro REACT seguiu por um 1 ano cerca de 5 mil pacientes de alto risco cardiovascular, sendo quase 70% em prevenção secundária. O perfil dos pacientes indica equilíbrio entre os sexos e, dentre os fatores de risco, HAS e dislipidemia foram os mais comuns (presentes em mais de 70% dos pacientes). Em cerca de 20% dos pacientes em prevenção secundária não foi identificada a prescrição de antiplaquetário, e o uso combinado de antiplaquetário, estatina e IECA em toda a população de alto risco foi de 28,3% na avaliação basal para 24,2% em 1 ano. O risco de eventos cardiovasculares maiores em 1 ano foi de 5,46 por 100 pessoas-ano, e os três fatores mais importantes associados a esses eventos foram inerentes à condição clínica do paciente: idade, prevenção secundária e nefropatia diabética.

O grupo incluído no REACT representa um grupo heterogêneo, mas que está de acordo com o conceito atual de prevenção cardiovascular, em que, mais importante do que classificar um indivíduo como portador de DM, HAS ou dislipidemia, é caracterizá-lo em termos de seu risco cardiovascular. A publicação com resultados parciais do REACT feita em 2013^15^ tinha incluído 2.403 pacientes e analisados 2.364 após análise de qualidade dos dados basais. Na presente análise, foram acrescidos 2.673 pacientes, o que totalizou 5.076 arrolados ao final do estudo (4.975 pacientes elegíveis para análise). Na publicação atual, além de o tamanho amostral ser mais que o dobro da amostra da publicação anterior, foi incluída a informação de avaliação prospectiva em 12 meses.^15^ Dessa forma, além de maior precisão na avaliação dos dados basais, foi possível incluir informações sobre a evolução desses pacientes. Havia carência de dados do seguimento em 12 meses de uma grande população contemporânea de pacientes de alto risco cardiovascular, pois mesmo em grandes estudos internacionais que incluíram a America Latina, como o REACH,^18^ o tamanho amostral de pacientes do nosso continente neste estudo,^18^ representou menos da metade dos casos incluídos no REACT.

Em termos de prescrição de terapias baseadas em evidência para redução do risco cardiovascular, identificou-se que terapias bem estabelecidas, como antiplaquetário para prevenção secundária, não foram prescritas para parcela significativa dessa população de alto risco. Em registros internacionais de pacientes de alto risco,^19 - 21^ foi identificada grande variabilidade na adesão terapêutica e no controle dos fatores de risco. No estudo REACT, mesmo com 90% dos pacientes sendo acompanhados em centros cardiológicos, foram identificadas, ainda, lacunas importantes no controle do risco cardiovascular. Com relação à prescrição médica, além de parcela significativa de não adesão na avaliação basal, no seguimento de 12 meses houve redução absoluta de aproximadamente 4% na prescrição combinada de antiplaquetário, estatina e IECA. Essas diferenças demonstram a necessidade da elaboração de estratégias para melhor controle dos fatores de risco com maior prescrição a terapias baseadas em evidência na população brasileira.^22^

O acompanhamento de 12 meses no estudo REACT permitiu a análise da taxa de eventos cardiovasculares maiores e dos seus principas preditores. Os fatores de maior associação identificados foram relacionados à condição do paciente, tais como idade, prevenção secundária e nefropatia, e estão de acordo com conceitos estabelecidos previamente em estudos internacionais.^21 , 23 , 24^ Tendo em vista tais achados, reitera-se a necessidade de ter a prevenção de doença cardiovascular e de doença renal como prioridades nas políticas de saúde pública. Rastreamento e controle adequado de fatores de risco como HAS, dislipidemia e DM são fundamentais nessa estratégia de prevenção primária de doença cardiovascular. No REACT, 10,3% dos pacientes sem diagnóstico prévio de HAS ou DM apresentaram níveis de pressão arterial e/ou glicemia dentro dos parâmetros patológicos. Dessa forma, além da busca de modelos eficientes de melhora da adesão das recomendações baseadas em evidência,^22^ há necessidade de melhorar a identificação dessas condições de risco na população e trabalhar de forma conjunta em seu controle. Isso se deve ao fato de que, embora a terapia baseada em evidência reduza o risco de eventos, a taxa de eventos ainda será mais elevada no grupo de prevenção secundária de forma independente de outras variáveis. Essa abordagem conjunta sistemática reforça o conceito de que os esforços preventivos não são apenas pelos riscos atribuíveis à elevação de fatores isolados, como a pressão arterial ou o colesterol sérico, mas pela ação nos múltiplos fatores, interferindo no risco absoluto global de cada indivíduo.

### Limitações do Estudo

Embora o convite tenha sido aberto para centros interessados no Brasil, as regiões Norte, Nordeste e Centro-Oeste tiveram representação proporcionalmente baixa. Além disso, os centros participantes eram, em sua maioria, cardiológicos e com estrutura de pesquisa clínica, e os participantes incluídos de forma voluntária. Desse modo, os resultados podem não ser aplicáveis a populações que não se enquadrem nessas características (p. ex., unidades de saúde com menos recursos, especialmente nas regiões Norte, Nordeste e Centro-Oeste). De qualquer maneira, mesmo em locais com condições mais favoráveis, foram identificadas lacunas relevantes na aplicação das práticas baseadas em evidência. Outras limitações incluem: outros possíveis fatores associados a eventos cardiovasculares, visto que não foram coletados como variáveis socioeconômicas e culturais dos pacientes; dados de desfecho clínico não foram adjudicados e não foram obtidos em 12 meses em 407 pacientes. Entretanto, a avaliação de desfecho clínico em estudos observacionais pragmáticos habitualmente é realizada por notificação do médico investigador, sem utilização de um comitê específico para adjudicação, e o REACT representaria um cenário mais próximo à forma de identificação de eventos que ocorre na prática clínica real. Com relação ao seguimento de 12 meses, tendo em vista que as perdas de dados ocorreram em momentos distintos, as análises foram realizadas por modelo de Cox e, por consequência, os pacientes foram censurados no último contato registrado como forma de minimizar as variações na duração de seguimento. Finalmente, a avaliação da adesão terapêutica se baseou na prescrição médica e não foram coletados dados sobre a elegibilidade, sobre a real utilização das terapias prescritas e sobre as principais barreiras para a prescrição e a utilização das terapias. Dessa maneira, os resultados do REACT refletem a adesão geral do médico em termos de prescrição de terapias baseadas em evidência, porém sem informação da utilização efetiva dessas terapias.

## Conclusão

Em um grande estudo prospectivo de pacientes de alto risco cardiovascular, foram identificadas falhas na prescrição de terapias baseadas em evidência acima do que seria esperado em registros internacionais, e tais falhas se intensificaram durante o seguimento de 1 ano. Identificou-se também uma taxa de eventos cardiovasculares acima de 5% ao ano nos pacientes incluídos já em prevenção secundária, a qual foi preditora independente de risco, assim como idade e nefropatia. Essas informações poderão ser utilizadas no desenvolvimento de projetos de melhoria de qualidade assistencial e de outras políticas de assistência à saúde, a fim de reduzir o risco dos eventos cardiovasculares na população brasileira.
