# Supplementary material for: Avaliação do Seguimento de 1 Ano dos Pacientes Incluídos no Registro da Prática Clínica em Pacientes de Alto Risco Cardiovascular (REACT)
Source: Arq Bras Cardiol. 2020 Dec 7;116(1):108–16. [Article in Portuguese] doi: 10.36660/abc.20190885 (PMC8159499; doi:10.36660/abc.20190885)
Supplement: Translation

# Translation: Evaluation of 1-Year Follow-up of Patients Included in the Registry of Clinical Practice in Patients at High Cardiovascular Risk (REACT)

**Authors:** Pedro Gabriel Melo de Barros e Silva, Otavio Berwanger, Dalton Bertolim Precoma, Margaret Assad Cavalcante, José Fernando Vilela-Martin, Estêvão Lanna Figueiredo, Renato Delascio Lopes, Luiz Carlos Bodanese, Jorge Ilha Guimarães, Jadelson Pinheiro de Andrade, Angelo Amato Vincenzo de Paola, Marcus Vinicius Bolivar Malachias, Luiz Alberto Piva e Mattos, Fernando Bacal, Oscar Pereira Dutra

**Affiliations:** 1 Brasil Instituto de Pesquisa HCor, Sao Paulo, SP - Brasil; 2 Brasil Hospital Samaritano Paulista, Sao Paulo, SP - Brasil; 3 Brasil Hospital Israelita Albert Einstein, Sao Paulo, SP - Brasil; 4 Brasil Pontificia Universidade Católica do Paraná - Escola de Medicina, Curitiba, PR - Brasil; 5 Brasil Sociedade Hospitalar Angelina Caron – Cardiologia, Campina Grande do Sul, PR - Brasil; 6 Brasil Universidade do Oeste Paulista (Unoeste), Presidente Prudente, SP – Brasil; 7 Brasil Hospital Regional de Presidente Prudente, Presidente Prudente, SP - Brasil; 8 Brasil Faculdade de Medicina de São José do Rio Preto (FAMERP), São José do Rio Preto, SP - Brasil; 9 Brasil Departamento de Hipertensão Arterial da Sociedade Brasileira de Cardiologia, Rio de Janeiro, RJ - Brasil; 10 Brasil Hospital Lifecenter, Belo Horizonte, MG - Brasil; 11 EUA Duke University Hospital, Durham, North Carolina - EUA; 12 Brasil Hospital São Lucas, Porto Alegre, RS - Brasil; 13 Brasil Sociedade Brasileira de Cardiologia, Rio de Janeiro, RJ - Brasil; 14 Brasil Hospital da Bahia, Salvador, BA - Brasil; 15 Brasil Universidade Federal de São Paulo Escola Paulista de Medicina, São Paulo, SP - Brasil; 16 Brasil Faculdade de Ciências Médicas de Minas Gerais, Belo Horizonte, MG - Brasil; 17 Brasil Instituto de Hipertensão Arterial - Diretoria Clínica, Belo Horizonte, MG - Brasil; 18 Brasil Rede D’or de Hospitais, São Paulo, SP - Brasil; 19 Brasil Universidade de São Paulo Faculdade de Medicina Hospital das Clínicas Instituto do Coração, São Paulo, SP - Brasil; 20 Brasil Instituto de Cardiologia – Fundação Universitária de Cardiologia do Rio Grande do Sul, Porto Alegre, RS – Brasil

**Keywords:** Cardiovascular Diseases, Risk Factors, Prescription Drugs, Multicenter Studies as Topic, Medical Record Linkage

## Abstract

**Background:**

In clinical practice, there is evidence of failure to prescribe evidence-based therapies for patients at high cardiovascular risk. However, in Brazil, data on 1-year outcomes of these patients remain insufficient.

**Objectives:**

To describe the use of evidence-based therapies and the occurrence of major cardiovascular outcomes and their major predictors in a 12-month follow-up of a Brazilian multicenter registry of patients at high cardiovascular risk.

**Methods:**

This prospective observational study documented the outpatient clinical practice of managing patients over 45 years of age and of high cardiovascular risk in both primary and secondary prevention. Patients were followed-up for 1 year, and the prescription of evidence-based therapies and the occurrence of major cardiovascular events (myocardial infarction, stroke, cardiac arrest, and cardiovascular death) were assessed. P-values < 0.05 were considered statistically significant.

**Results:**

From July 2010 to August 2014, a total of 5076 individuals were enrolled in 48 centers, 91% of the 4975 eligible patients were followed-up in cardiology centers, and 68.6% were in secondary prevention. At 1 year, the concomitant use of antiplatelet agents, statins, and angiotensin-converting enzyme inhibitors reduced from 28.3% to 24.2% (p < 0.001). Major cardiovascular event rate was 5.46%, and the identified predictors were age, patients in secondary prevention, and diabetic nephropathy.

**Conclusions:**

In this large national registry of patients at high cardiovascular risk, risk predictors similar to those of international registries were identified, but medical prescription adherence to evidence-based therapies was inferior and significantly worsened at 1 year. (Arq Bras Cardiol. 2020; [online].ahead print, PP.0-0)

## Introduction

Cardiovascular diseases are usually manifestations arising from an arterial atherosclerotic substrate.^1 - 4^ Together, they affect more than 4% of the global population and their acute complications, known as cardiovascular events, are the leading cause of death and disability in both men and women worldwide.^2 - 4^ In Brazil, as in other developing countries, the frequency of those diseases continues to increase over the years, which reinforces the need for a better understanding of the outcomes of those patients in clinical practice.^2 - 7^

Despite the high morbidity and mortality, several strategies to reduce the risk of complications in those patients have been developed.^8 - 12^ Among the options, patients at high cardiovascular risk may benefit from antithrombotic (antiplatelet) therapies, statins, and angiotensin-converting enzyme inhibitors (ACEIs) or angiotensin receptor blockers (ARBs).^8 - 12^ However, the use of those therapies in clinical practice has proved to be insufficient, especially in developing countries.^13 - 15^ In Brazil, previously reported partial data from the Registry of Clinical Practice in Patients at High Cardiovascular Risk (REACT) showed that the combined use of antiplatelet agents, statins, and ACEIs was identified in only 34% of this population.^15^ Despite the relevance of those data, there are limitations in the analysis because information on medical prescription adherence to evidence-based therapies was collected in a cross-sectional fashion and changes in prospective follow-up have not been reported yet. Furthermore, there remains the need to identify the actual expected event rate and the predictors associated with such events in a Brazilian population of individuals at high cardiovascular risk.

The present study aimed to assess, in patients at high cardiovascular risk treated at Brazilian centers over 12 months, the proportion of those continuously receiving interventions with proven benefit and the factors associated with late clinical outcomes, particularly major cardiovascular event rate during follow-up.

## Methods

The REACT registry is a project to document the actual care of patients at high cardiovascular risk in centers across all Brazilian regions, including both public and private hospitals as well as primary health care units.

### Study Design and Implementation

The REACT registry is a Brazilian Society of Cardiology (SBC) project whose operation was conducted by the HCor Research Institute (IP-HCor) and whose methods were reported elsewhere.^15 , 16^ Briefly, this is an observational, prospective, multicenter study whose inclusion of patients occurred voluntarily from July 2010 to August 2014 in 48 health care facilities that included both public and private hospitals as well as primary health care units. All 5 Brazilian regions were covered with the following distribution of participating centers: Southeast (45.8%), North (6.3%), Northeast (14.6%), South (29.2%), and Midwest (4.2%). For the selection of participating centers, open invitations were sent to interested centers by the SBC and the coordinating center (IP-HCor). The study was initiated after approval by the relevant Research Ethics Committee, and data were collected after individual patient consent was obtained. Nationwide data from the cross-sectional analysis that documented the clinical practice of managing patients at high cardiovascular risk have been reported elsewhere.^15^ Additionally, longitudinal follow-up of these patients at 6 and 12 months had the following objectives: to measure medical prescription adherence to recommended evidence-based therapies, to evaluate the occurrence of major cardiovascular events, and to identify their respective predictors.

### Study Participants

Briefly, study participants should be over 45 years of age and have at least one of the following factors:^15 , 16^ 1) any clinical evidence of arterial disease (coronary artery, cerebrovascular, or peripheral artery disease); 2) diabetes mellitus (DM); 3) 3 cardiovascular risk factors, except DM: hypertension, smoking, dyslipidemia, age over 70 years, diabetic nephropathy, family history of coronary artery disease, asymptomatic (subclinical) carotid artery disease. The first group had known arterial disease and consisted of patients considered to be in a stage of secondary prevention regardless of having other inclusion criteria. Other participants were considered as primary prevention with DM (second inclusion criterion) or without DM (those included only by the third inclusion criterion). Because this was a clinical practice study with pragmatic criteria, the exclusion criteria were refusal to provide informed consent, a psychiatric or neurocognitive condition that prevented obtaining reliable clinical data (at the investigators’ discretion), and life expectancy less than 6 months.

### Study Procedures and Analyzed Variables15,16

Data were collected at admission for baseline data (index visit) and also at two follow-up visits at 6 and 12 months to measure medical prescription adherence to recommended evidence-based therapies and to assess occurrence of major cardiovascular events. These follow-up visits could be conducted in person at the centers or by telephone. Because this was a pragmatic study, the identification of comorbidities (e.g., hypertension, dyslipidemia) could be performed as follows: report by patient, use of (antihypertensive, lipid-lowering) drugs, or at the investigators’ discretion (in the latter, the centers were advised to follow the recommended diagnostic criteria adopted in the current SBC guidelines). Data on drug prescriptions were collected to assess medical prescription adherence to evidence-based recommendations. The evidence-based therapy regimen that was considered in the REACT registry was consistent with current guidelines.^8 - 12^ No data were collected on the effective use of drugs by patients.

### Study Outcomes

As described in previously reported REACT methods,^16^ the primary outcome was related to prescription of interventions with proven benefit (e.g., aspirin, statins, ACEIs) and impact on late clinical outcomes. Late clinical outcomes included myocardial infarction, stroke, cardiac arrest, and overall and cardiovascular mortality.^15 , 16^ These outcomes were reported by the investigator, with no participation of an independent event adjudication committee.

### Statistical Analysis

The distribution of continuous variables was assessed for normality using histograms. Normally distributed continuous variables were described as mean ± standard deviation. Categorical variables were described as absolute and relative frequencies, and proportions were compared by the chi-square test or the Fisher-Freeman-Halton exact test. Independent predictors of combined events (death, myocardial infarction, cardiac arrest, or stroke) were identified using Cox proportional hazards models, as data on the dates of the events were collected. This predictor analysis was initially performed in a univariate fashion to assess the following factors: age, sex, history of coronary artery disease, previous acute myocardial infarction, history of stroke/transient ischemic attack, history of peripheral artery disease, DM, hypertension, diabetic nephropathy, smoking, asymptomatic carotid artery disease, and combined use of antiplatelet agent, statin, and ACEI at baseline. Variables with p-value < 0.15 were included in a multivariate analysis. Reported p-values are two-tailed, and p < 0.05 was considered statistically significant in the final analyses. The assumptions of proportionality for each predictor and global variable were assessed using standardized Schoenfeld residuals.^17^ Generalized estimating equation (GEE) models were used to assess drug therapy over time. All analyses were conducted using the software R, version 3.6.1.

## Results

Between July 2010 and August 2014, 5076 patients were recruited in this national registry; however, excluding patients without eligibility and baseline data, 4975 patients remained for analysis, 91% of whom were followed-up at cardiology centers ( Table 1 ). For 407 patients (8.2%), obtaining 12-month follow-up data was not possible (loss to follow-up).

**Table 1:** – Baseline characteristics

Baseline characteristics	Total (n = 4975)
Age; mean ± SD	65.4 ± 10 (n = 4975)
Sex (male)	2614/4975 (52.5%)
Ethnicity	
White	3422/4975 (68.8%)
Black	571/4975 (11.5%)
Yellow (Asian)	76/4975 (1.5%)
Brown	900/4975 (18.1%)
Red (native Brazilian)	6/4975 (0.1%)
Type of center	
Cardiology	4505/4950 (91%)
Neurology	7/4950 (0.1%)
Vascular surgery	3/4950 (0.1%)
Endocrinology	114/4950 (2.3%)
Internal medicine	99/4950 (2%)
Primary care	222/4950 (4.5%)
Prevention	
Primary	428/4975 (8.6%)
Primary with DM	1135/4975 (22.8%)
Secondary	3412/4975 (68.6%)
BMI; mean ± SD	28.5 ± 5.2 (n = 4959)
BMI ≥ 25	3660/4959 (73.8%)
CAD	2867/4975 (57.6%)
Previous acute myocardial infarction	1510/4975 (30.4%)
Stroke	710/4975 (14.3%)
Peripheral artery disease	799/4975 (16.1%)
DM	2814/4975 (56.6%)
Multiple risk factors (at least 3)	3057/4975 (61.4%)
Hypertension	4451/4975 (89.5%)
Dyslipidemia	3638/4975 (73.1%)
Diabetic nephropathy	406/4975 (8.2%)
Age > 70 years	1700/4975 (34.2%)
Current smoking	515/4975 (10.4%)
Family history of CAD	2478/4975 (49.8%)
Asymptomatic carotid artery disease	605/4975 (12.2%)
Blood pressure	
Systolic	132.3 ± 19.7 (n = 4975)
Diastolic	79.5 ± 11.4 (n = 4975)
Laboratory tests	
Total cholesterol (mg/dL)	178 ± 58.5 (n = 3041)
LDL-cholesterol (mg/dL)	99.6 ± 39 (n = 2834)
HDL-cholesterol (mg/dL)	45.4 ± 14.4 (n = 2996)
Triglycerides (mg/dL)	159.8 ± 116.3 (n = 3049)
Blood glucose (mg/dL)	126.7 ± 55.2 (n = 3327)
Glycated hemoglobin (%)	7.2 ± 2.1 (n = 1953)
Creatinine (mg/dL)	1.1 ± 0.8 (n = 3305)

*BMI: body mass index; CAD: coronary artery disease; DM: diabetes mellitus; HDL: high-density lipoprotein; LDL: low-density lipoprotein; SD: standard deviation.*

### Baseline Characteristics

The patients’ clinical profile showed that mean age was 65.4 (± 10), 52.5% were male, and 68.6% were patients in secondary prevention ( Table 1 ). Coronary artery disease was the most common diagnosis of established cardiovascular disease and was found in almost 60% of the sample ( Table 1 ).

## Medical Prescription Adherence to Evidence-based Therapies

Among the patients included in the study, 74.6% used antiplatelet agents, 72.2% used statins, and 42.5% used ACEIs ( Table 2 ). The percentage varied according to the inclusion criterion and was higher in the secondary prevention group, in which the use of antiplatelet agents and the use of statins was close to 80% ( Table 2 ). Among the patients with history of myocardial infarction, 73.8% received beta-blockers at baseline. At follow-up, the concomitant use of antiplatelet agents, statins, and ACEIs reduced from 28.3% to 24.2% (p < 0.001), and the most evident reduction was found in ACEI users ( Figure 1 ).

**Table 2:** – Use of therapies for cardiovascular prevention and control of risk factors according to population characteristics

	Primary (n = 428)	Primary with DM (n = 1135)	Secondary (n = 1733)	Secondary and DM (n = 1679)	Total (n = 4975)	p
**Drug (baseline)**						
Antiplatelet agent	225/428 (52.6%)	731/1135 (64.4%)	1403/1733 (81%)	1354/1679 (80.6%)	3713/4975 (74.6%)	< 0.001
Statin	276/428 (64.5%)	720/1135 (63.4%)	1347/1733 (77.7%)	1249/1679 (74.4%)	3592/4975 (72.2%)	< 0.001
ACEI	171/428 (40%)	519/1135 (45.7%)	758/1733 (43.7%)	787/1679 (46.9%)	2235/4975 (44.9%)	0.043
Combination	64/428 (15%)	263/1135 (23.2%)	527/1733 (30.4%)	554/1679 (33%)	1408/4975 (28.3%)	< 0.001
Beta-blocker (patient with AMI)			607/816 (74.4%)	507/694 (73.1%)	1114/1510 (73.8%)	-
Thiazide diuretic (patients with hypertension)	174/387 (45%)	555/1038 (53.5%)	496/1481 (33.5%)	642/1545 (41.6%)	1867/4451 (41.9%)	< 0.001
**Control of risk factors (baseline)**						
Glycated hemoglobin						
< 7%	146/150 (97.3%)	321/655 (49%)	361/408 (88.5%)	292/740 (39.5%)	1120/1953 (57.3%)	< 0.001
7% to 8%	1/150 (0.7%)	144/655 (22%)	22/408 (5.4%)	150/740 (20.3%)	317/1953 (16.2%)	
≥ 8%	3/150 (2%)	190/655 (29%)	25/408 (6.1%)	298/740 (40.3%)	516/1953 (26.4%)	
Blood glucose						
< 100 mg/dL	185/284 (65.1%)	137/838 (16.3%)	664/1074 (61.8%)	236/1131 (20.9%)	1222/3327 (36.7%)	< 0.001
100 to 125 mg/dL	90/284 (31.7%)	268/838 (32%)	342/1074 (31.8%)	310/1131 (27.4%)	1010/3327 (30.4%)	
≥ 126 mg/dL	9/284 (3.2%)	433/838 (51.7%)	68/1074 (6.3%)	585/1131 (51.7%)	1095/3327 (32.9%)	
Blood pressure						
< 130/80 mm Hg	274/428 (64%)	582/1135 (51.3%)	1066/1733 (61.5%)	904/1679 (53.8%)	2826/4975 (56.8%)	< 0.001
130/80 to 139/89 mm Hg	97/428 (22.7%)	322/1135 (28.4%)	432/1733 (24.9%)	466/1679 (27.8%)	1317/4975 (26.5%)	
≥ 140/90 mm Hg	57/428 (13.3%)	231/1135 (20.4%)	235/1733 (13.6%)	309/1679 (18.4%)	832/4975 (16.7%)	
LDL-cholesterol						
< 50 mg/dL	1/269 (0.4%)	40/712 (5.6%)	53/939 (5.6%)	93/914 (10.2%)	187/2834 (6.6%)	< 0.001
50 to 69 mg/dL	25/269 (9.3%)	97/712 (13.6%)	145/939 (15.4%)	173/914 (18.9%)	440/2834 (15.5%)	
≥ 70 mg/dL	243/269 (90.3%)	575/712 (80.8%)	741/939 (78.9%)	648/914 (70.9%)	2207/2834 (77.9%)	

*P-value (chi-square test) < 0.05 indicates that preventive therapy/risk factor are dependent on the population characteristic. ACEI: angiotensin-converting enzyme inhibitor; AMI: acute myocardial infarction; DM: diabetes mellitus; LDL: low-density lipoprotein.*

**Figure 1:** – Prescription of cardiovascular prevention therapies according to follow-up time. To compare the continuity of drug prescription between follow-ups and baseline, a generalized estimating equation (EEG) model was adjusted for binary data to account for dependence between observations. ‡ p-value < 0.001; comparison between follow-up and baseline. † p-value < 0.01; comparison between follow-up and baseline. * p-value < 0.05; comparison between follow-up and baseline. ACE: angiotensin-converting enzyme; AMI: acute myocardial infarction.

## Control of Risk Factors

Overall, 16.7% of patients had blood pressure ≥ 140 x 90 mm Hg. In baseline laboratory assessment, glycated hemoglobin was < 7% in 47.5% of diabetic patients, with control being more frequent in primary prevention patients. Low-density lipoprotein (LDL)-cholesterol level was > 70 mg/dL in 76.6% of patients, and > 90% of secondary prevention patients had LDL-cholesterol > 50 mg/dL. Among the patients without previous diagnosis of hypertension and/or DM, 17.9% (94/524) had blood pressure ≥ 140 x 90 mm Hg, 3.6% (77/2161) had fasting blood glucose ≥ 126 mg/dL, and 4.1% (88/2161) had glycated hemoglobin ≥ 6.5%. In a combined fashion, 10.3% (247/2392) of the patients without previous diagnosis of hypertension or DM had pathological levels of blood pressure or blood glucose.

Guidance for nonpharmacological measures was reported in about 80% of prescriptions, being similar in both primary and secondary prevention groups for smoking cessation, but higher in primary prevention group for physical activity and cardioprotective diet.

## Clinical Outcomes

Overall (either cardiovascular or not) mortality at 12 months was 4.92%; this was higher in the Northeast region (9.33%; 95% CI 6.1%-12.6%) followed by the Midwest (8.6%; 95% CI 3.0%-14.1%), South (4.9%; 95% CI 3.7%-6.1%), and Southeast (4.3%; 95% CI 3.5%-5.1%) regions. The analysis of the North region was compromised by low inclusion (99 patients) with 30% loss to follow-up, with report of only 1 death (1.5%; 95% CI 0.0%-4.3%).

Major cardiovascular event rate in the total population was 5.46 per 100 patient-years in the secondary prevention group ( Figure 2 ), and the predictors identified for cardiovascular events were age, secondary prevention, and diabetic nephropathy ( Table 3 ).

**Figure 2:** – One-year event rate according to inclusion criterion. AMI: acute myocardial infarction; DM: diabetes mellitus; HR: hazard ratio.

**Table 3:** – Predictive factors for cardiovascular risk. Univariate and multivariate analyses

Variables	Univariate analysis		Multivariate analysis	
	HR [95% CI]	p-value	HR [95% CI]	p-value
Age (1-year increment)	1.036 [1.025; 1.047]	< 0.001	1.035 [1.024; 1.046]	< 0.001
Sex (male)	1.123 [0.900; 1.401]	0.303	-	-
History of CAD (yes)	1.686 [1.329; 2.139]	< 0.001	1.324 [0.989; 1.772]	0.059
Previous AMI (yes)	1.672 [1.338; 2.090]	< 0.001	1.515 [1.155; 1.988]	0.003
History of stroke/TIA (yes)	1.738 [1.335; 2.263]	< 0.001	1.481 [1.132; 1.938]	0.004
History of PAD (yes)	1.951 [1.520; 2.503]	< 0.001	1.651 [1.271; 2.143]	< 0.001
DM (yes)	1.191 [0.951; 1.492]	0.127	1.227 [0.967; 1.557]	0.093
Hypertension (yes)	0.829 [0.593; 1.159]	0.272	-	-
Diabetic nephropathy (yes)	1.826 [1.324; 2.518]	< 0.001	1.438 [1.021; 2.025]	0.037
Smoker (yes)	0.950 [0.656; 1.376]	0.785	-	-
Asymptomatic carotid artery disease (yes)	1.008 [0.724; 1.404]	0.963	-	-
Combined drugs (yes)*	1.083 [0.852; 1.377]	0.513	-	-

*Combined drugs: combined use of antiplatelet agent, statin, and angiotensin-converting enzyme inhibitor at baseline. AMI: acute myocardial infarction; CAD: coronary artery disease; HR: hazard ratio; PAD: peripheral artery disease; TIA: transient ischemic attack; DM: diabetes mellitus.*

## Discussion

The REACT registry followed-up for 1 year approximately 5000 patients at high cardiovascular risk, almost 70% of whom were in secondary prevention. The patients’ profile shows a balance between male and female, and hypertension and dyslipidemia were the most common risk factors (found in > 70% of patients). Antiplatelet prescription was not identified in about 20% of secondary prevention patients, and the combined use of antiplatelet agent, statin, and ACEI in the entire high-risk population ranged from 28.3% at baseline to 24.2% at 1 year. The risk of major cardiovascular events at 1 year was 5.46 per 100 patient-years, and the three most important factors associated with such events were inherent to patient clinical status: age, secondary prevention, and diabetic nephropathy.

Although heterogeneous, the group of patients included in the REACT registry is in line with the current concept of cardiovascular prevention, in which characterizing individuals in terms of cardiovascular risk is more important than classifying them as having DM, hypertension, or dyslipidemia. Previously reported partial results of the REACT registry from 2013^15^ had included 2403 patients and analyzed data from 2364 after baseline data quality analysis. In the present analysis, 2673 patients were added to the previous sample, leading to a total of 5076 participants at the end of the study (4975 patients eligible for analysis). In the current report, in addition to the sample being more than double the previously reported sample, prospective data on 12-month follow-up were included.^15^ Thus, in addition to allowing greater precision in the assessment of baseline data, this report included data on patient outcomes. There were limited data on 12-month follow-up from a large contemporary population of patients at high cardiovascular risk because, even in large international studies that included Latin America such as the REACH trial,^18^ the sample size of patients from our continent in this study,^18^ represented less than half of the cases included in the REACT study.

Regarding prescribed evidence-based therapies to reduce cardiovascular risk, this study found that well-established therapies such as antiplatelet agents for secondary prevention were not prescribed for a significant portion of the high-risk population. In international registries of high-risk patients,^19 - 21^ there was great variability in adherence to therapy and control of risk factors. In the REACT study, even with 90% of patients being followed-up at cardiology centers, important gaps in the control of cardiovascular risk were identified. Regarding medical prescription, in addition to a significant proportion of nonadherence at baseline, there was an absolute reduction of approximately 4% in the combined prescription of antiplatelet agent, statin, and ACEI at 12-month follow-up. These differences demonstrate the need to develop strategies for a better control of risk factors with greater prescription of evidence-based therapies in the Brazilian population.^22^

Twelve-month follow-up in the REACT study allowed an analysis of the rate of major cardiovascular events and their major predictors. The factors with stronger association were related to patient status, such as age, secondary prevention, and nephropathy, and are consistent with previously established concepts in international studies.^21 , 23 , 24^ In view of such findings, having primary cardiovascular and kidney disease prevention as a priority in public health policies is required. Adequate screening and control of risk factors such as hypertension, dyslipidemia, and DM are crucial in this primary cardiovascular disease prevention strategy. In the REACT registry, 10.3% of patients without previous diagnosis of hypertension or DM had blood pressure and/or blood glucose levels within pathological parameters. Thus, in addition to the search for efficient models to improve adherence to evidence-based recommendations,^22^ there is a need to improve the identification of these risk conditions in the population and work together to control them. This is because, although evidence-based therapy reduces the risk of events, event rate will remain higher in the secondary prevention group regardless of other variables. This joint systematic approach reinforces the concept that preventive efforts are not related only to the risks attributable to the elevation of isolated factors, such as blood pressure or serum cholesterol, but also to the action of multiple factors, affecting the overall absolute risk of each individual.

### Study Limitations

Although the invitation was open to interested centers across Brazil, the North, Northeast, and Midwest regions had a proportionally low representation. Additionally, the participating centers were mostly cardiology centers and had a structure for clinical research, and the participants were included voluntarily. Thus, the results may not be applicable to populations that do not fit these characteristics (e.g., health care facilities with fewer resources, especially in the North, Northeast, and Midwest regions). Nonetheless, even in facilities with more favorable conditions, relevant gaps were identified in the application of evidence-based practices. Another limitation is related to possible factors associated with cardiovascular events, as patient socioeconomic and cultural variables were not collected and clinical outcome data were not adjudicated, with missing 12-month data from 407 patients. However, clinical outcome review in pragmatic observational studies is usually conducted by investigator’s report, without any specific adjudication committee, and the REACT registry represents a scenario closer to the identification of events in actual clinical practice. Regarding the 12-month follow-up, considering that data losses occurred at different time points, analyses were performed using the Cox model and, therefore, patients were censored at the last recorded contact to minimize variations in follow-up duration. Finally, adherence to therapy was assessed based on medical prescriptions and no data were collected on eligibility, on the actual use of prescribed therapies, and on the main barriers to the prescription and use of therapies. Thus, the REACT results reflect physicians’ overall adherence in terms of prescribing evidence-based therapies, but without data on the actual use of these therapies.

## Conclusion

In a large prospective study of patients at high cardiovascular risk, failures in the prescription of evidence-based therapies were higher than what is expected in international registries, and these failures increased during the 1-year follow-up. A cardiovascular event rate > 5% per year was also identified in patients included as secondary prevention, which was an independent predictor of risk, as well as age and nephropathy. These findings can be used in the development of projects to improve quality of care and other health care policies in order to reduce the risk of cardiovascular events in the Brazilian population.
